# Keeping up the beat of Kleefstra syndrome

**DOI:** 10.1186/s13052-026-02207-8

**Published:** 2026-02-07

**Authors:** Giulia Bruna Marchetti, Federica Gaudioso, Camilla Meossi, Michela Mura, Carlo Agostoni, Cristina Gervasini, Valentina Massa, Laura Pezzoli, Maria Iascone, Donatella Milani

**Affiliations:** 1 Fondazione IRCCS Ca’ Granda Ospedale Maggiore Policlinico di Milano, Milan, Italy; 2 Molecular Medicine, IRCCS Fondazione Stella Maris, Pisa, Italy; 3 Department of Clinical and Community Sciences, Università degli Studi di Milano, Milan, Italy; 4 Department of Health Sciences, Università degli Studi di Milano, Milan, Italy; 5 “Aldo Ravelli” Center for Neurotechnology and Experimental Brain Therapeutics, Università Degli Studi di Milano, Milan, Italy; 6 Laboratorio di Genetica Medica, ASST Papa Giovanni XXIII, Bergamo, Italy

**Keywords:** Long QT, Kleefstra syndrome, EHMT1, Mendelian disorder of epigenetic machinery

## Abstract

Long QT syndrome (LQTS) is a rare and potentially life-threatening arrhythmia characterized by delayed repolarization on electrocardiogram (EKG) evaluation. Although LQTS is primarily associated with defects in ion channels, in approximately 20% of cases the genetic cause remains unknown. Kleefstra syndrome (KS), a rare neurodevelopmental disorder caused by alterations in the *EHMT1* gene, has been associated with various cardiac abnormalities, including structural defects and arrhythmias. Here we report the second case of LQT in a patient with KS, strengthening the association between these two conditions. Although rare in KS, LQT may represent a potentially life-threatening condition that requires careful monitoring. Further, we present a detailed clinical case and a literature review on cardiac rhythm abnormalities in KS, highlighting the importance of EKG monitoring in these patients. Further studies are needed to clarify the link between *EHMT1* and cardiac arrhythmias and to establish the potential role of *EHMT1* in the epigenetic modulation of cardiac pacemakers. These insights may have important implications in management of patients with KS and other Mendelian disorders of epigenetic machinery (MDEMs).

**Supplementary Information:**

The online version contains supplementary material available at 10.1186/s13052-026-02207-8.

## Introduction

Heart development represents a complex and tightly regulated process, which errors give rise to the most frequent malformations among newborns [1]. Emerging evidence suggests that chromatin state patterns, together with structural heart defects and early embryonic events might play a role in cardiac arrhythmias etiology [2]. Epigenetic machinery critical role during human development is also pivotal for heart development [3]. Long QT syndrome (LQTS) represents a rare but potentially life-threatening arrhythmia characterized by delayed repolarization on electrocardiogram (EKG) evaluation. Although LQTS is primarily related to defects in ion channel activity, the genetic cause of this condition remains unknown in approximately 20% of cases [4].

Methylation represents a major epigenetic mechanism associated with gene silencing and dependent on the activity of several proteins [5]. The impairment of Euchromatic Histone Methyltransferase 1 (*EHMT1*, MIM #*607001*, on chromosome 9q34.3) results in Kleefstra syndrome (KS, MIM #610253), one condition in the complex and overlapping spectrum of Mendelian disorders of epigenetic machinery (MDEMs). KS is a rare neurodevelopmental disorder, characterized by moderate-to-severe intellectual disability, hypotonia, autistic-like features and, often, distinctive facial traits. Seizures, sleep and behavioral anomalies are common, but signs and symptoms can potentially involve any organ and tissue. Reported cardiac abnormalities in KS include both structural defects and/or arrhythmia, but the full spectrum is yet to be elucidated [6]. Rots and colleagues have recently estimated that 31% of KS patients display an abnormality of heart morphology and 12% a rhythm disturbance. Only one case of KS with LQT was reported [7].

Here we report a further case of LQT in a KS patient, strengthening the association between these two conditions. A deeper understanding of the link between KS and heart arrhythmias, particularly LQT, is crucial not only to adequately monitor these patients but also to prevent cardiac events. Furthermore, investigating the connection between *EHMT1* and heart pacemaker in larger cohorts may provide valuable insights into mechanisms underlying cardiac physiology and development.

## Case report

The patient is the third child of a healthy non-consanguineous couple. Pregnancy was complicated by Hashimoto thyreopathy and polyhydramnios. He was born at 36 weeks from cesarean section, birth parameters were normal (Weight: 3100 g, Lenght: 49.5 cm, OFC: 34 cm). Apgar score was 9/10. Neonatal Jaundice was treated with phototherapy. After birth, hypotonia and feeding difficulties were noticed and developed into a “slow” deglutition ability. The remaining neonatal history was uneventful, with the only exception of urinary tract infection at 3 months of age.

Psychomotor development was severely impaired: he reached seated position at 10–12 months and walked independently at 28. The first words were pronounced at 48 months of age. Last neuropsychiatric evaluation through WISC IV at the age of 11 years, confirmed a moderate degree intellectual disability (IQ:40). His neurological picture was enriched by aspecific EEG and brain MRI alterations. Particularly a thin corpus callosum, reduced thickness of frontal gyri, incomplete opercularization of sylvan valleys. Further clinical details included: hypermetropia, inguinal hernia surgically treated and dental caries.

At physical examination at the age of 11 years, growth parameters were within normal range (weight: 36.5 kg, height: 144 cm, OFC: 52.5 cm). Brachycephaly, thick eyebrows, hypertelorism, wide nasal root and midface hypoplasia were noticed. Three hypopigmented macules with Maine cost margins were detected at skin evaluation.

From a cardiological point of view, the patient underwent a first at rest EKG evaluation at the age of 7 years with the detection of a slight elonged QTc (441 ms). He repeated the cardiological evaluation at the age of 11 years and was thereafter diagnosed with an exercise induced LQT. In detail, at Holter EKG, QTc was above normal values for under stress heart rate (up to 150 bpm). Also straight ST line or notched T wave were observed during exercise. Basal EKG always remained in normal range. No structural heart defect was detectable through ultrasound examination. He reported no family history for heart disorders.

His diagnostic iter passed through karyotype, performed both on blood and skin cells, CGH array and multiplex ligation probe amplification (MLPA) of *RAI1* gene (MIM #* 607642), all resulted normal.

In this child, KS diagnosis was reached through Next Generation Sequencing (NGS) analysis of a panel of genes related to intellectual disability and autism spectrum disorders with the evidence of a missense *de novo* variant c.3577G > A, p.(Gly1193Arg) in *EHMT1* gene. The variant was classified as pathogenic.

A targeted NGS analysis was carried out to exclude secondary diagnosis. The LQTS test detected no pathogenic variants.

### Methods

Once informed consent was obtained, DNA was extracted from leukocytes using standard procedure and PCR-free whole genome sequencing (Illumina, PE 2 × 150) was performed. A virtual panel of 13 genes associated with cardiac arrhythmias were bioinformatically selected for analysis, namely: *CACNA1C* (NM_000719.7), *CALM1* (NM_006888.6), *CALM2* (NM_001743.6), *CALM3* (NM_005184.4), *CASQZ* (NM_001232.4), *KCNH2* (NM_000238.4), *KCNJ2* (NM_000891.3), *KCNQ1* (NM_000218.3), *RYR2* (NM_001035.3), *SCN5A* (NM_001099404.2), *SLC4A3* (NM_005070.4), *TECRL* (NM_001010874.5) and *TRDN* (NM_006073.4).

Variants were classified on the basis of ACMG guidelines [8] and following modifications [9, 10]. Genes selection and variants interpretation were performed according to disease-specific guidelines [11, 12].

We also reviewed published literature searching for rhythm abnormalities in KS. We collected data from two different articles, both collecting data from wide cohorts of KS patients [6, 7]. Also, we included two further cases of KS patients of our cohort displaying EKG anomalies. Data from the three KS patients of our cohort were retrieved in the contest of the Drop by Drop research project, dedicated to create an Italian registry of KS patients and to test possible therapies using in vitro models. The project up to date recruited 45 KS patients and pivots on two other centers in North Italy, the Genetic Laboratory of the ASST Papa Giovanni XXIII, Bergamo where diagnostic data have been revised, and the Department of Health sciences of the University of the Studies, in Milan. The project was approved by the local Ethics Committee and conducted according to Good Clinical Practice and Helsinki Declaration.

**Table 1:** Clinical data of our patient and of previous reports of EKG anomalies in Kleefstra syndrome

Source	Patients	EHMT1 alteration (GRCh38)	Inher.	AgeDia	Other variants	EKG abn.	LQT	SVA	VA	SHD	Specify
OurCo	Reported	c.3577G > C p.Gly1193Arg	Dn	5		Y	Y			N	
OurCo	2	chr9:(137666340–138059695)x1	Dn	31		UnspArr	N			N	
OurCo	3	c.642 + 2T > A	Unk.	0		Y	N	TPSV		Y	ASD
[1; 2]	KS110068, pt Radboudumc	g.137,728,442 C > T, c.736 C > T, p.(Arg246Ter)	Unk	25	*CYP2C19*	Y	N	PAF		N	
[1; 2]	KS110012, pt Radboudumc	g.137,798,894 C > T, EHMT1, c.2587 C > T, p.(Gln863Ter)	Unk	18	CYP2C9*2, Het	Y	N	SVT		N	
[1; 2]	KS110002, pt Radboudumc	g.137776794dup, EHMT1, c.1968dup, p.(Gln657AlafsTer41)	Unk			Y	N	PSVT		N	
[1; 2]	KS110009, pt Radboudumc	NC_000009.12, g.pter(203862_534475), del(137507220_138125938)	Unk	22	Chr9: (p)*DOCK8*, *KANK1*, (q) *CACNA1B*	Y	N		VT	Y	Valve insufficiency
[1]	GenIDA	Unknown	Unk.	< 29		Y	N	AF		NR	
[1]	GenIDA	Unknown	Unk.	< 19		Y	N	AET		NR	
[1]	Patient 1	16 kb intragenic duplication, encompassing exons 5–6a	Pat.	23		Y	N	PAF, Aflu, PACs	NSVT	N	
[1]	Patient 2	IVS22-1G > A c.3259G > A	Dn	25		Y	N	PAF		N	
[1]	Patient 3	c.3462–10 C-G, (IVS 24–10 C-G)	Unk	17		Y	N	SVT		N	
[2]	KS110013	chr9: g.137,815,945 A > G, c.3259–2 A > G	Dn	Unk		Y	Y			N	
[2]	KS110060	chr9: g.137574569_138212068del	Unk	Unk		Y	N	SinT		Y	PF, VSD
[2]	KS110061	chr9: g.137813531G > T, c.3180 + 1G > T	Unk	Unk		Y	N	PAF		Y	LVH
[2]	KS110064	chr9: g.137574569_138212068del	Unk	Unk		Y	N	AF		N	
[2]	KS110071	chr9: g.137170000_138360000del	Unk	Unk		HP	N			Y	ASD type II, PuSt
[2]		chr9: g.137814468G > A, c.3218G > A, p.Cys1073Tyr	Dn	Unk	16q22.3, del 149 kb, mat	Y	N	SVT		Y	PuSt
[2]	KS110001	chr9: g.137814434T > G, c.3184T > G, p.Cys1062Gly	Dn	Unk		HP	N			N	
[2]	KS110036	chr9: g.137779715T > C, c.2273T > C, p.Leu758Pro	ParMos	Unk		Y	N	SVT		Y	ASD
[2]	KS130012	chr9: g.137834391_137834402del, c.3583_3594del, p.Val1195_Phe1198del	ParMos	Unk		UnspArr	N			N	
**TOT**						**22**	**2**	**14**	**2**	**7**	

1 = Vasireddi et al., 2023; 2 = Rots et al., 2022. Below abbreviations used: Abnormality = abn.; Age at diagnosis= AgeDia; Atrial ectopic tachycardia = AET; atrial flutter = AFlu; Atrial Septal Defect = ASD; Deletion = del; heart palpitations = HP; Heterozygous = Het; Left ventricular hypertrophy = LVH; Maternal = Mat; Paternal = Pat.; PAF= Paroxysmal atrial fibrillation; Paroxysmal SVT = PSVT; Parental Mosaicism= ParMos; Patient = pt; Perimembranous Foramen = PF; Premature Atrial Contractions= PACs; Pulmonary Stenosis= PuSt; Sinus tachycardia= SinT; Structural heart disease = SHD; Supraventricular SVA Supraventricular tachycardia (SVT); Unknown = Unk; Unspecified arrhythmia; UnspArr Ventricular Septal Defect = VSD; Ventricular tachycardia = VT

## Discussion

To our knowledge, this is the second reported case of LQT in KS [7], although rhythm anomalies have been sporadically described in KS (See Table 1). This is the first report deepening the molecular investigation of LQT in a patient diagnosed with KS. In the presented case, a targeted molecular analysis excluded a second genetic diagnosis underlying LQT. These novel data reinforces the hypothesis that the association between KS and LQTs might not be coincidental and suggesting a role of EHMT1 in rhythm disturbances. It is to note that epigenetic mechanisms such as post-translational histone modification, non-coding RNA, and DNA methylation in cardiovascular development are attracting scientific interest [13]. In particular, genes involved in the regulation of histone 3 lysine 4 (H3K4) have been related to several congenital heart diseases. Histone 3 lysine 9 (H3K9) methylation complex recognizes *EHMT1* as a key player [14]. The reported case and the negative ruslts of genetic deepening performed in this child, together with existing literature [6, 7], strengthens the link between EHMT1 and the proper functioning of cardiac pacemaker, as already established for other methyltransferase.

We collected data from two previous paper in order to gain a wider point of view of EKG abnormalities associated to KS. Furthermore, we included cardiological data of three KS patients displaying EKG anomalies and visited in our Medical Center (see Table 1). In total we collected 22 patients with KS and an anomaly in EKG. Main rhythm anomalies reported in KS involve supraventricular structures (14/22), including supraventricular tachycardia (6/14) and atrial fibrillation (4/14). In only 2 patients a ventricular arrhythmia was reported. Rots and colleagues also reported one further patient displaying LQT, possibly related to the splicing pathogenic variant identified in *EHMT1* [7]. From a clinical point of view, no further cardiological insights are available on this patient, who mainly displays a neurocognitive impairment (intellectual disability, autism, behavioral problems, and seizures are reported) [7].

The present report strengthens the association of KS to rhythm anomalies, in particular to LQT, which might represent a life-threatening condition if underestimated and not adequately monitored. Our aim is to emphasize the importance of EKG monitoring in KS, as already depicted in specific management guidelines for KS [15]. Despite the relative rarity of cardiac pace impairment in KS (with an estimated prevalence as high as 8% [6], that aligns with the data retrieved from our cohort), it is extremely important to bear in mind that, also in adult age, these subjects may be prone to develop arrhythmias. Clinicians’ awareness about these uncommon manifestations in KS should be particularly high in reason of the severe limitations these patients may overcome in communicating any symptom [6]. In particular, the possible association of KS to long QT, although still not definitely established, is certainly an important *caveat* for clinicians and cardiologists dealing with KS.

In a broader perspective, it is interesting to note that KS might represent the first condition among MDEMs associated not only to morphological defects in heart development [16] but also to anomalies in its physiology. In most MDEMs, actually, once ultrasound examination rules out congenital heart defects, no further cardiological assessments might be considered. Despite the limitations given by the retrospective nature of our study, the present report, together with the review of EKG anomalies in KS and the strong phenotypical overlap that characterizes MDEMs, gives important insights into the best cardiological monitoring routine in this group of rare disorders.

## Conclusions

The present report strengthens the association between LQT and KS. Data from a literature review furtherly underscore the importance of EKG monitoring in KS, providing valuable insights in management of patients affected by MDEMs.

## Supplementary Information

Below is the link to the electronic supplementary material.

Supplementary Material 1

## Data Availability

The datasets used and analysed in the current study are available from the corresponding author on reasonable request.
