# Supplementary material for: Keeping up the beat of Kleefstra syndrome
Source: Ital J Pediatr. 2026 Feb 7;52:38. doi: 10.1186/s13052-026-02207-8 (PMC12977797; doi:10.1186/s13052-026-02207-8)
Supplement: Supplementary file 1 — Supplementary Material 1 [file 13052_2026_2207_MOESM1_ESM.odt]

Reviewer 3: 

The case is well described, but is the second report of LQT in Kleefstra syndrome: I believe that the association between these conditions may be coincidental.
In my opinion the authors should focus on LQT which was right now observed only in a minority of KS patients mentioning that this patient confirm that heart rhythm problems are frequent in KS and probably, causally related with molecular pathogenesis of the disease 

Answer: 

Dear Reviewer, 

Following your suggestions we have attempted to emphasize in the discussion of our work the idea that the present case, together with genetic test performed to exclude other possible genetic causes, confirm that heart rhythm problems are frequent in KS and possibly recognize in EHMT1 defects a causative role. 

Please find attached the revised manuscript. 

Thank you for your help, 

Dr. Giulia Bruna Marchetti
